# Emergence in Western African Countries of MDR-TB, Focus on Côte d'Ivoire

**DOI:** 10.1155/2013/426709

**Published:** 2013-09-04

**Authors:** Euloge Ekaza, Raymond Kouassi N'Guessan, Adèle Kacou-N'Douba, N'Guetta Aka, Jacquemin Kouakou, Françoise Le Vacon, Fréderic Altare, Gilles Potel, Marie-France de-La-Cochetiere

**Affiliations:** ^1^Département de Bactériologie-Virologie, Institut Pasteur de Côte d'Ivoire, 01 BP 490 Abidjan 01, Cote D'Ivoire; ^2^Thérapeutiques Cliniques et Expérimentales des Infections, Département des Maladies Infectieuses UFR de Médecine, 1, rue Gaston Veil, 44035 Nantes Cedex, France; ^3^Programme National de Lutte Contre la Tuberculose, 05 BP 1054 Abidjan 05, Cote D'Ivoire; ^4^Biofortis Research, Biofortis SAS, Mérieux Nutriscience Company, 3 route de la Chatterie, 44800 Saint-Herblain, France; ^5^INSERM-Unité 892—CNRS 6299, 8 quai Moncousu, BP 70721, 44007 Nantes Cedex 1, France; ^6^INSERM, Thérapeutiques Cliniques et Expérimentales des Infections, Département des Maladies Infectieuses UFR de Médecine, 1, rue Gaston Veil, 44035 Nantes Cedex, France

## Abstract

Tuberculosis (TB) is responsible for a high mortality rate (2.5%) worldwide, mainly in developing countries with a high prevalence of human immunodeficiency virus (HIV). The emergence of multiresistant strains of TB poses an extreme risk for TB outbreaks and highlights the need for global TB control strategies. Among Western African countries, Côte d'Ivoire (CI) represents a specific example of a country with great potential to prevent TB. Specifically, CI has a promising healthcare system for monitoring diseases, including vaccination programs. However, military and political conflict in CI favors the spread of infectious diseases, TB being among the most devastating. Compilation of the studies identifying common causes of TB would be extremely beneficial for the development of treatment and prevention strategies. Therefore, the purpose of this comprehensive review is to evaluate the epidemiology of TB in CI, describe the factors involved in pathogenesis, and suggest simple and applicable prevention strategies.

## 1. Introduction

Tuberculosis (TB) is considered among a group of diseases which poses the greatest global health risk. Each year 8.8 million new cases of TB are diagnosed worldwide, resulting in 1.4 million deaths [1, 2]. Forty percent of the individuals infected with TB live in South-East Asia and another 24% are located in Sub-Saharan Africa, but with the most important number of deaths (>50% of death worldwide) [1]. Côte d'Ivoire (CI), a Sub-Saharan country with a promising healthcare system for monitoring diseases, including vaccination programs, has great potential to prevent infectious disease and is the focus of this work. The aim of this review is to understand the growing impact of TB in CI and support the development of prevention and treatment strategies. CI spans 322,462 square kilometers in Western Africa and is home to approximately 22 million people [3]. The population of CI grew at a rate of 2.78% and the average life expectancy was estimated at 52 years in 2009 [3]. Further, CI has a high immigration rate (26%) due to both geographical location and a historical reputation, placing CI at the center of economic and cultural exchange [4]. Poverty and HIV infection are among the major factors contributing to spread of TB [5]. CI has the highest prevalence of HIV/AIDS in Western Africa, occurring at a rate of 3.4% and an additional 2.4% persons considered living with HIV (PLHIV) [6] in 2009. Further, the most common cause of death in PLHIV is TB [7, 8]. In 2010, 23210 TB cases were reported in CI, 0.26% of all TB cases diagnosed worldwide, including active disease cases (65% of notified TB cases in CI) and HIV coinfected cases (24% of notified TB cases in CI) [1]. Environmental conditions and cultural habits have a considerable amount of influence on the development of TB. Civil war, for example, is frequent and often leads to the destruction of healthcare systems. The consequences of this destruction are poor patient access to healthcare facilities and a lack of compliance when medications are prescribed, hence, microbial resistance develops [9]. Further, several *Mycobacterium tuberculosis* strains have been recently reported as resistant to locally available treatments [10–12]. Therefore, the purpose of this review is to describe the current state of TB in CI, understand the emergence of resistance, and discuss the management of this devastating disease.

## 2. Tuberculosis

TB is caused by members of the *Mycobacterium tuberculosis* complex, closely related group of organisms which infect humans (*M. tuberculosis*, *M. africanum*, and *M. canettii*) and rodents (*M. microti*), whereas other members of this complex have a wide host spectrum (e.g., *M. bovis* and* M. bovis *BCG vaccine strain) [13]. Infected respiratory aerosol is the main route of transmission.

**Figure 1:**
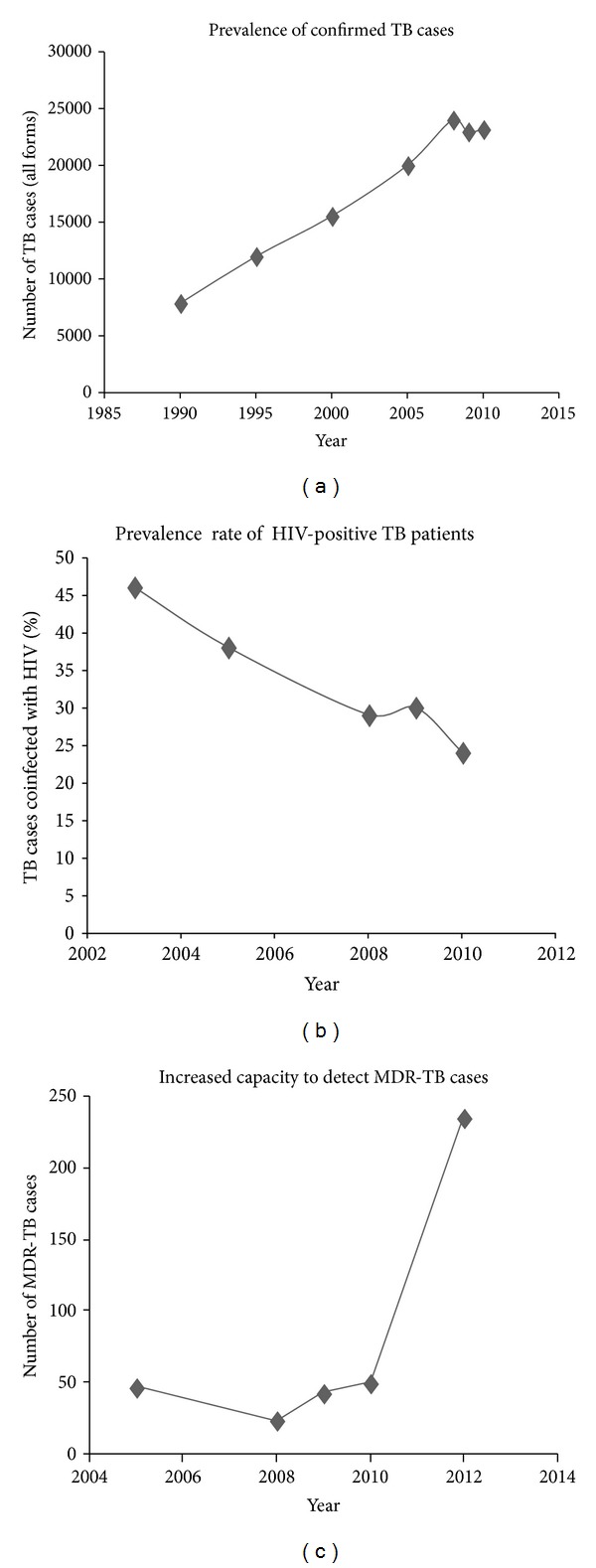
The evolution of confirmed TB, MDR-TB, and TB/HIV coinfected cases in Côte d'Ivoire. (a) Each year TB cases are notified. The total number of diagnosed TB cases has increase since 1990, (b) the number of coinfected TB/HIV patients and deaths as result of TB in PLHIV, and (c) the raise may be due to the targets, implementation of LPAs, and EXPAND TB in general.

In 2006, the prevalence of TB in CI exceeded the number of cases in the entire region of Sub-Saharan Africa [14]. TB is most commonly diagnosed in young economically productive males (15–45 years of age) [15, 16]. TB outbreaks are associated with impoverishments and in turn malnutrition. Since 2002, TB outbreaks in CI have been further exacerbated by massive emigration, resulting from armed conflict. Finally, the HIV/AIDS pandemic remains an essential factor in TB infection and the most common opportunistic infection occurring in PLHIV [7, 8, 17, 18]. TB and HIV, together, are a lethal combination, maximizing one anothers development. So, the decision to integrate HIV/AIDS with TB struggle was taken. The number of PLHIV increases with antiretroviral therapy (ART) and antituberculosis (Anti-TB) treatment; therefore, a link between the reduction in TB mortalities and a reduction in TB/HIV coinfection mortalities was observed. The prevalence of TB/HIV coinfection decreased from 46% in 2003 to 24% in 2010 (Figure 1(b)) as a result of (i) the experience of the national TB control program (NTP) in following-up patients, (ii) the implementation of TB/HIV coinfection control programs (Global Funds to Fight against AIDS, Tuberculosis, and Malaria (GFATM); United States President's Emergency Plan For AIDS Relief (PEPFAR)) by nongovernmental organizations (NGO), and (iii) the strategic planning and priorities of UNAIDS [1, 7]. In 2010, roughly 4,112 people coinfected with TB/HIV were treated for HIV infection, 26% were treated with ART regardless of their CD4-lymphocyte cell count [1], and 80% began preventive treatment with both cotrimoxazole/Isoniazid (H)) and ART [1, 19]. In CI, this prophylactic cocktail has significantly lowered the mortality rate of patients coinfected with TB/HIV [20]. However, the prevalence of HIV infection in patients diagnosed with TB is probably underestimated since the documentation of HIV infection among TB patients is a recent practice [14].

## 3. Multidrug-Resistant Tuberculosis: Epidemiological Evidence

### 3.1. Multidrug-Resistant TB

MDR-TB generally develops as a consequence of insufficient, irregular, or unsuitable treatment. MDR-TB is a worldwide health concern. Main cause of drug resistance in poor countries is closely related to lack of access to free or affordable healthcare services. In CI, management of TB cases may be globally improved; first-line drug therapy for TB after diagnosis is the combination of Rifampin (R)/Isoniazid (H)/Pyrazinamid (Z)/Ethambutol (E) for two months, followed by R/H treatment for four months (6 months total treatment). For all retreatment cases (relapse, failure, and defaulter) a second line of treatment is administered: two months of drugs R/H/Z/E/Streptomycin (S), followed by one month of R/H/Z/E, and finally five months R/H/E (8 months total treatment) [21]. These anti-TB drugs are available, free of charge, in all anti-TB centers (ATC) and diagnostic and treatment centers (DTC).

From ten years, in CI, first-line and second-line anti-TB drugs are usually available using mechanisms to prevent stockouts. The NTP receives every quarter the commands of processing centers. The validation of needs is made on the basis of the reports of results of treatment and screening general tendencies. The NTP translates command to PSP for a current need for 3 months and a reserve of 3 months every time. The anti-TB drugs are bought by PSP (CI budget) or GDF (Global TB Drug Facility from GFATM). Drugs (PSP or GDF) are centralized at PSP level which assures the centers provisioning according to their needs.

The MDR-TB strains are typically resistant to at least R and H. The extensively drug-resistant tuberculosis (XDR-TB) strains acquire resistance to R and H, fluoroquinolone, and any of the following injectable antibiotics: capreomycin, kanamycin, and amikacin [22]. Recent term “totally drug-resistant tuberculosis” (TDR-TB) was given by authors to MDR-TB strains resistant to all second-line drugs tested [10–12]. The term “TDR-TB” is not yet recognized by the WHO because data on the reproducibility and reliability of *in vitro* drug susceptibility testing (DST) for remaining second-line anti-TB drugs are either much more limited or have not been established [23]. New drugs are under development and their effectiveness against these TDR-TB strains has not yet been reported. According to WHO definitions, these cases are defined as XDR-TB.

To determinate the mechanism associated to isoniazid (H) resistance, a preliminary study on few *M. tuberculosis* strains had showed that Ser315Thr substitution in the *katG* gene was the main cause [24].

CI currently uses the (Directly Observed Therapy Shortcourse) DOTS strategy, recommended by the WHO, adopted in 1995 to reduce the rate of TB morbidities. DOTS remains at the heart of the Stop TB Strategy. This strategy combines five basic components: (1) political commitment with increased and sustained financing, (2) case detection through quality-assured bacteriology, (3) standardized treatment regimen directly observed by a healthcare worker or community health worker for at least the first two months, (4) an effective drug supply and management system, and (5) monitoring and evaluation system, and impact measurement of treatment results. In 2009, 79% of new TB cases were successfully treated and globally this percentage has been increasing since 1995. Tuberculosis usually infects the lungs but it can also infect other parts of the body, including kidneys, spine, and brain (extrapulmonary TB). Extrapulmonary TB (24% of all notified cases), smear-negative pulmonary TB (10% of all notified cases), and smear unknown (1% of all notified cases) have been reported in CI in 2009 [1]. In extrapulmonary and smear-negative pulmonary TB, the rate of therapeutic success was 75%, it was 64% in cases of relapses. Regardless of this therapeutic success, the number of newly diagnosed extrapulmonary TB cases continues to increase at an alarming rate [1, 25]. CI has implemented a routine surveillance program to track drug-resistant TB cases. The epidemiological state of TB in CI is characterized by an incidence rate of 191 new cases per 100000 inhabitants [26] and virtual stabilization of the MDR-TB notified cases between 2004 to 2011 [1, 25, 27, 28]. Two national surveys were conducted in 1995-1996 and in 2004 to assess primary resistance to anti-TB drugs [27, 29]. Indeed, the prevalence of MDR-TB among untreated TB patients was reduced from 5.3% in 1995-1996 [29] to 2.5% in 2004 [27]. Because before 2012, the process of renovation and equipment of TB laboratories for diagnostic capacity for TB and MDR-TB strains in the country were not at his term, TB labs in 2010 reported 50 MDR-TB cases in patients submitted for reprocessing [1]. Recent report showed, 235 MDR-TB cases among suspected cases of MDR-TB [30] (previously treated cases, and new cases living with a MDR-TB case) in 2012 (Figure 1(c)). The first explanation it is targets: in 2012, the bounce concerns all the reprocessing cases (failure, relapse; taken back) which are a NTP priority because these cases have more risk to develop high percentage of resistant strains. A study shows that the retreatment failure was the most predictive indicator for MDR-TB [31]. In it, add the chronic TB cases that we have since a few years and which with the strengthening were confirmed. In the past, suspected cases had no access to bacteriological diagnosis because of analysis costs. The war only could not explain it. Capacity to test for second-line drug resistance is ongoing. More than 50% of both pulmonary TB cases and MDR-TB cases with positive microscopy are detected annually in the district of Abidjan [32]. An understanding of the transmission dynamics of TB strains has been improved by rapid molecular genotyping techniques, which have resulted in the association of major lineages with geographic regions and ultimately linked to ancient human migration. They can allow a better response from health authorities and the implementation of measures for TB control. Very few studies have been conducted in CI. One of them included few strains (not representative of the situation) isolated from new cases and in one region of the country [33]. A recent study may suggest that *M. tuberculosis* complex strains among retreatment cases exhibit a low diversity, allowing to assume recent transmission and locally based infection [34]. 

## 4. Efforts to Combat TB and Prevention

**Figure 2:**
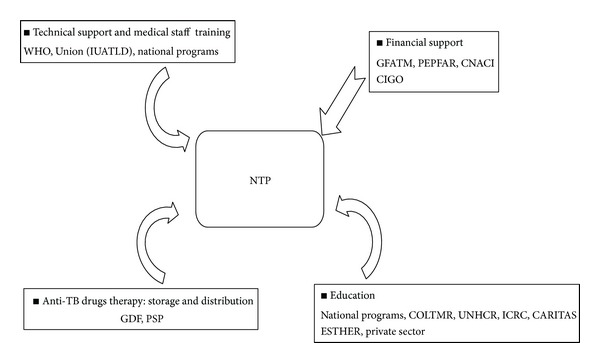
Major focus areas of the NTP in Côte d'Ivoire and supporting organizations. CNACI: National Antituberculosis Committee of Côte d'Ivoire; COLTMR: Tuberculosis and Respiratory Illness Organizational Collective; ESTHER: French Group for In-Network Hospital Treatment Solidarity; GDF: Global Tuberculosis Drug Facility; GFATM: Global Fund to control AIDS, Tuberculosis and Malaria; CIGO: the Côte d'Ivoire Government; ICRC: International Committee of the Red Cross; NTP: National Tuberculosis control Program; PEPFAR: President's Emergency Plan For AIDS Relief; PSP: Public Health Pharmacy; UNHCR: United Nations High Commissioner for Refugees; Union or precedently IUATLD: International Union Against Tuberculosis and Lung Disease; and WHO: the World Health Organization. CI depends heavily on external organizations for financial aid to combat the spread of TB.

Programs focused on early diagnosis are essential in combating TB infection. The implementations of adapted treatments and strict hygiene procedures have been shown to reduce the risk of transmission [35]. The limited access to and general lack of quality healthcare in the conflict zones contribute to the spread of TB infection. In an effort to contain TB infection, programs for coverage and the training of staff at every level of the sanitary pyramid have been implemented in CI. Programs have also been established to provide free and consistent access to anti-TB drugs [36]. The support of a number of organizations has further benefited the capability of the NTP to combat TB infection (Figure 2). In 2010, 140 DTC and 16 ATC including 1642 health workers were functioning. The TB prevention in CI hospitals and clinics is structured in three tiers: administrative/organizational (standard protection measures for individual), environmental ventilation measures to reduce infectious particle concentration in air (direction of natural ventilation or correct working locations), and personal protection (e.g., hygiene when we present respiratory symptoms and use of individual protective respiratory apparatus). The implementation of these three strategies has largely prevented TB infection among healthcare providers. The details of these strategies outlined in the national guide for the control of TB transmission in CI healthcare services [36].

**Figure 3:**
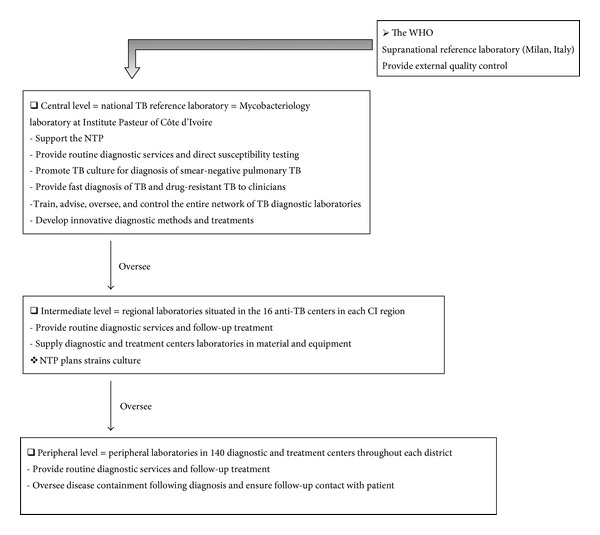
Organization of diagnostic tuberculosis laboratories in Côte d'Ivoire. CIGO has constructed a network of laboratories for TB diagnosis, treatment, and monitoring. This network is organized in three tiers: central level, national laboratory for TB; intermediate level, anti-TB center laboratories; and peripheral level, diagnostic and treatment center laboratories.

NTP has a network of microscopy laboratories, which provide the bacteriological followup of suspected TB cases and ultimately diagnoses. This network consists of 3 levels: a national TB reference laboratory (NTRL), regional laboratories, and peripheral laboratories (Figure 3). Civil war has deterred these efforts. However, the incidence rate for TB has been steadily increasing since 1990, regardless of the slight decline in 2009 and 2010 suggesting innovative prevention programs need to be employed [1, 25, 28]. Screening has been extensively implemented in the privately-funded clinics, prisons, and HIV/AIDS clinics. The implication of communities in methods to combat TB infection has contributed to treatment success, which increased from 76% of new cases in 2009 to 79% in 2010 [1, 25].

### 4.1. Perspectives

A new strategic plan for TB prevention and treatment (2012–2016) has been implemented since the end of civil war. This plan aims to (i) provide accurate diagnoses and improve access effective treatment, (ii) provide follow-up evaluations and epidemiological surveillance of TB/HIV coinfection and MDR-TB, (iii) employ treatment and prevention strategies in sectors without strategies in place through the Ministry of Health, and (iv) promote TB research. It is expected that complete execution of this strategy will result in the detection of 70% of pulmonary TB cases and a treatment success rate of 85% by 2016. Meeting these objectives will partially fulfill the 2015 CI Millennium Development Goal decreasing TB incidence and mortality rates.

### 4.2. Financial Load

The American emergency plan for AIDS relief (PEPFAR) provided 17 million USD, over 5 years (Figure 2), which are mainly used for those patients that are coinfected with TB/HIV. Results from a study conducted in 2006 suggest that the average cost of TB treatment per patient, using DOTS, was 121 USD per year. In the case of failure or treatment interruption, the average cost of second-line anti-TB drug therapy per patient increased to 464 USD for 6 months of treatment [37]. Thus, the cost of treatment (first-line) and followup for the 21,691 patients diagnosed with TB in 2010 is roughly 2 million USD [1]. Despite the support of a number of external organizations the number of patients infected with MDR-TB remains high in CI, and treatment is a national financial burden. In 2010, diagnosis of MDR-TB cases was estimated at 2.5% (~542 cases) of all TB diagnoses, roughly equivalent to 251,488 USD in treatment expenses (6 months of treatment), excluding the laboratory costs associated with diagnosis [1]. The costs associated with the follow-up clinic visits, including second-line drug therapy for MDR-TB, represent approximately 0.04% (~2.2 million USD for 6 months of treatment) of the entire budget for CI.

*New Diagnosis Tools and Laboratories Reinforcement.* It is estimated that with direct examination of sputum, more than half of contagious TB cases are not detected. This leads to propagation of TB agent in the community (disease transmission) [38]. CI NTP is eligible to participate in the EXPAND-TB project which provides laboratory support for TB diagnosis with the goal of strengthening the diagnostic capacity for TB and MDR-TB strains. So, new algorithm using molecular line probe assays (LPAs) for the diagnostic of TB and drug susceptibility testing from smear-positive sputum and from strains was implemented in the country [39].

At the request of the Ministry of Health in CI, and working with partners, FIND engaged in a project to strengthen TB diagnostic services by providing a long-term, on-site consultant to work in close collaboration with the NTRL and NTP. In parallel, the NTRL and another laboratory have been renovated, with the creation of both biosafety level BSL-2+ and BSL-3 (for NTRL) facility that meets the requirements recommended by WHO for handling liquid TB culture. TB solid culture and drug susceptibility testing (DST) have been implemented, with EQA provided by the WHO Supra National Reference Laboratory of the South Africa Medical Research Council located in Pretoria. Subsequently, liquid culture for TB and DST, along with molecular assays that can provide information on drug resistance in addition to detecting *M. tuberculosis* complex, has been implemented. Reporting on isolation and contamination rates for solid culture on Lowenstein Jensen media and TB liquid culture on the BACTEC MGIT 960 TB System has been available since 2012.

## 5. Discussion and Conclusion

TB is responsible of approximately 2.5% of deaths worldwide each year, mainly in developing countries [2]. In low-income nations, this disease is spreading with devastating consequences. Recent reports confirm that a belt of MDR-TB exists in Eastern Europe, Western Europe, and Central Asia [10, 40–42]. Many countries, in the same area, still lack reliable MDR-TB surveillance systems. Therefore, this finding is highly worrisome. The mismanagement of nondrug-resistant TB cases, which leads to drug resistance, is a continuing problem everywhere, even in high-income European countries with low TB incidence [43]. The number of incurable XDR-TB cases is on the rise, in several countries. Suitably for these cases, and especially for those experiencing treatment failures, which is difficult and has obvious logistical, ethical, and economic implications [44]. Efforts to understand the evolution of MDR-TB in African countries, India, and the Russian Federation are also of critical importance [12, 42]. This review provides a picture of the epidemiology and a description of the factors which contribute to the pathogenesis of TB, in a country with high prevalence of TB. In short, this problem has two dimensions: social issues (e.g., civil war) and the emergence of antibiotic-resistant microorganisms. The increasing incidence of this disease in CI, in association with HIV infection, requires innovative strategies and community education programs to improve quality of life. 

TB moved back to 40% in 20 years, but, anyhow, there will be more than 2 million new cases of MDR-TB between 2011 and 2015 [45]. The surge of MDR-TB cases and the lack of financial resources to manage them could compromise advances in TB treatment and prevention. Strategies to diagnose and treat MDR-TB must be expanded quickly to reach the international goal, which is to diagnose and treat 85% of MDR-TB cases by 2015 [45]. A major obstacle hindering this goal is the poor quality and capacity of national laboratories. In poor countries, where TB is endemic, bacteriological confirmation of TB is carried out in most cases after Ziehl-Neelsen staining. Culture and drug-susceptibility testing are not available; consequently, information for detection, followup, and management of antibiotic resistance in *M. tuberculosis* cannot be provided by laboratories. Using LPAs in routine diagnostic algorithms has several advantages. Besides its good sensibility and specificity for detection of resistances (Rifampicin alone or in combination with Isoniazid) in isolates of *M. tuberculosis* and in smear-positive sputum specimen [46, 47] it reduces time of the detection of MDR-TB, cost (between 30% and 50% less than conventional DST methods), and the need for sophisticated and costly laboratory infrastructure [46–48]. For these reasons, the use of LPAs has been recommended by the WHO [47, 48]. UNITAID, which contributes to the EXPAND-TB project, supports the improvement of national laboratories [1].

Mechanisms used by NTP to prevent stockouts are good and work correctly. When drug-procurement procedures are centralized through GDF, drug costs are lower and are available [43]. Very few studies have been conducted on availability of drugs and stockouts in neighboring countries. In general, they used the same procedures but, the differences are at stocks management level [49, 50]. In CI, stocks and centers provisioning are made by PSP, a structure which has experience in medicine management as it supplies all of the country health centers with medicine. In the neighboring countries, it is NTP which manages stocks and has to face the medicine quality control problems [49, 50]. With the support of many organizations, CI has implemented a healthcare system which includes disease monitoring and vaccination programs in an effort to contain infectious diseases. The HIV/AIDS pandemic and 10 years of civil war have reduced the services provided by healthcare centers, strongly aggravating morbidities associated with infectious diseases. Regional emigration favors the interruption of TB treatment and increases active TB transmission. Healthcare systems have been severely disrupted, particularly in the North and the West regions of the country; 80% of healthcare units in these areas have closed and 85% of the healthcare workers have left. Therefore, both disease monitoring systems and immunization programs have been disrupted [14]. Nonetheless, the country is a member of the Millennium Development Goal (MDGs), which aims to reduce the prevalence of TB by 50% (relative to the prevalence rates documented in 1990), detect at least 80% of the symptomatic cases, and cure 85% of them. At CI level, the important indicators are the number of notified cases, the coinfection TB/HIV, the rate of death and lost sight, rate of cures (cured and treatment completed), and so forth. From this point of view, the national aims are not reached yet, but there is progress in certain regions, in the prevalence of MDR-TB cases, in the never treated patient.

The CI government (CIGO) has made a tremendous effort in preventing the spread of infectious disease, including a sanitary plan inherited from the colonial era and a recently established healthcare system. The healthcare system in CI is largely supported by the government, with a rapidly expanding private sector, and collaborate with traditional (indigenous) medicine. CI setup a national program for promoting traditional medicine, which is the primary form of healthcare for more than 80% of population due to poverty and inaccessible healthcare centers in rural areas. In the case of the TB, the care covers all the country with TB laboratories network that provide routine diagnostic services and follow-up treatment. Generally, the population is informed that anti-TB drugs are available and free. Usually, it is the delay of diagnosis that is problematic, because, most often, patients go to the traditional medicine. But, studies on plants extracts suggest that they got an effect against mycobacteria [51]. So, it should be necessary to strengthen the collaboration with the traditional phytotherapists to allow them to participate in the process of diagnosis. Furthermore, this collaboration will increase population's confidence in healthcare centers.

Within the framework of the administrative decentralization policy, regions have a degree of autonomy to interfere with sanitary planning and care. Thus, this healthcare system has future potential. Further, CI is home to a Pasteur Institute, part of the international network of Pasteur Institutes, and the WHO collaborating center.

CIGO has made a commitment to the goal of universal healthcare coverage [52]. Recommendations have been given by the WHO, health worker, and community, including (i) use of generic medicines, although the purchasing power of the CI population is considerably low, (ii) financial and technical incentives for healthcare workers, and (iii) inspections to evaluate healthcare centers. Ensuring the functionality of both BSL-2+ and 3 level laboratories and the use of advanced mycobacteria detection methods, including culturing systems (e.g., MGIT960 system) and molecular techniques (e.g., HAIN Lifescience technology) will rapidly and drastically improve both, the detection and treatment of TB in CI.

## Abbreviations

AIDS: Acquired immunodeficiency syndrome
ART: Antiretroviral therapy
ATC: Antituberculosis centers
BSL: Biosafety level
CI: Côte d'Ivoire
CIGO: The Côte d'Ivoire Government
CNACI: National Antituberculosis Committee of Côte d'Ivoire
COLTMR: Tuberculosis and Respiratory Illness Organizational Collective
DOTS: Directly Observed Therapy Shortcourse
DST: Drug susceptibility testing
DTC: Diagnostic and treatment centers
E: Ethambutol
EQA: External Quality Assessment
ESTHER: French Group for In-Network Hospital Treatment Solidarity
EXPAND-TB: Expanding Access to New Diagnostics for Tuberculosis
FIND: Foundation for innovation new diagnostics
GDF: Global Tuberculosis Drug Facility
GDP: Gross Domestic Product
GFATM: Global Funds to Fight against AIDS, Tuberculosis and Malaria
H: Isoniazid
HIV: Human immunodeficiency virus
ICRC: International Committee of the Red Cross
LPAs: Molecular line probe assays
MDGs: The Millennium Development Goal
MDR-TB: Multidrug-Resistant Tuberculosis
MGIT: Mycobacterial Growth Indicator Tube
NGO: Nongovernmental organizations
NTP: National tuberculosis control program
NTRL: National tuberculosis reference laboratory
PEPFAR: United States President's Emergency Plan For AIDS Relief
PLHIV: Persons considered Living with HIV
PSP: Public health pharmacy
R: Rifampin
S: Streptomycin
TB: Tuberculosis
TDR-TB: Totally drug-resistant tuberculosis
UNAIDS: United Nations Program on HIV/AIDS
UNHCR: United Nations High Commissioner for Refugees
Union: precedently IUATLD (International Union Against Tuberculosis and Lung Disease)
UNITAID: Unit aid initiative
WHO: World Health Organization
XDR-TB: Extensively drug-resistant tuberculosis
Z: Pyrazinamide.
